# Raw Bowl Tea (Tuocha) Polyphenol Prevention of Nonalcoholic Fatty Liver Disease by Regulating Intestinal Function in Mice

**DOI:** 10.3390/biom9090435

**Published:** 2019-09-01

**Authors:** Bihui Liu, Jing Zhang, Peng Sun, Ruokun Yi, Xiaoyan Han, Xin Zhao

**Affiliations:** 1 Chongqing Collaborative Innovation Center for Functional Food, Chongqing University of Education, Chongqing 400067, China; 2 Chongqing Engineering Research Center of Functional Food, Chongqing University of Education, Chongqing 400067, China; 3 Chongqing Engineering Laboratory for Research and Development of Functional Food, Chongqing University of Education, Chongqing 400067, China; 4 College of Biological and Chemical Engineering, Chongqing University of Education, Chongqing 400067, China; 5 Environment and Quality Inspection College, Chongqing Chemical Industry Vocational College, Chongqing 401228, China

**Keywords:** raw Bowl tea, polyphenols, nonalcoholic fatty liver disease, mice, intestinal function

## Abstract

A high-fat diet-induced C57BL/6N mouse model of non-alcoholic fatty liver disease (NAFLD) was established. The effect and mechanism of Raw Bowl Tea polyphenols (RBTP) on preventing NAFLD via regulating intestinal function were observed. The serum, liver, epididymis, small intestine tissues, and feces of mice were examined by biochemical and molecular biological methods, and the composition of RBTP was analyzed by HPLC assay. The results showed that RBTP could effectively reduce the body weight, liver weight, and liver index of NAFLD mice. The serum effects of RBTP were: (1) decreases in alanine aminotransferase (ALT), aspartate aminotransferase (AST), alkaline phosphatase (AKP), total cholesterol (TC), triglyceride (TG), low density lipoprotein cholesterol (LDL-C), D-lactate (D-LA), diamine oxidase (DAO), lipopolysaccharide (LPS), and an increase of high density lipoprotein cholesterol (HDL-C) levels; (2) a decrease of inflammatory cytokines such as interleukin 1 beta (IL-1β), interleukin 4 (IL-4), interleukin 6 (IL-6), interleukin 10 (IL-10), tumor necrosis factor alpha (TNF-α), and interferon gamma (INF-γ); (3) a decrease the reactive oxygen species (ROS) level in liver tissue; and (4) alleviation of pathological injuries of liver, epididymis, and small intestinal tissues caused by NAFLD and protection of body tissues. qPCR and Western blot results showed that RBTP could up-regulate the mRNA and protein expressions of LPL, PPAR-α, CYP7A1, and CPT1, and down-regulate PPAR-γ and C/EBP-α in the liver of NAFLD mice. In addition, RBTP up-regulated the expression of occludin and ZO-1, and down-regulated the expression of CD36 and TNF-α in the small intestines of NAFLD mice. Studies on mice feces showed that RBTP reduced the level of *Firmicutes* and increased the minimum levels of *Bacteroides* and *Akkermansia*, as well as reduced the proportion of *Firmicutes*/*Bacteroides* in the feces of NAFLD mice, which play a role in regulating intestinal microecology. Component analysis showed that RBTP contained seven polyphenolic compounds: Gallic acid, (-)-epigallocatechin, catechin, L-epicatechin, (-)-epigallocatechin gallate, (-)-gallocatechin gallate, and (-)-epicatechin gallate (ECG), and high levels of caffeine, (-)-epigallocatechin (EGC), and ECG. RBTP improved the intestinal environment of NAFLD mice with the contained active ingredients, thus playing a role in preventing NAFLD. The effect was positively correlated with the dose of 100 mg/kg, which was even better than that of the clinical drug bezafibrate.

## 1. Introduction

Nonalcoholic fatty liver disease (NAFLD) is a multifactorial disease, associated with a complex living environment, heredity, and dietary habits. High calorie diets and prolonged inactivity contribute to weight gain by promoting the development of NAFLD [1]. The most common cause of NAFLD is systemic energy imbalance, with calorie intakes exceeding calorie consumption. Excess visceral adipose tissue contributes to ectopic fat in liver, skeletal muscle, and pancreas in the form of non-esterified fatty acids (NEFA) [2]. Accumulation of triglycerides in liver and blood further aggravate NAFLD. Both exercise and improved diet can reduce the risk of NAFLD [3]. 

Bowl Tea is a compacted tea with a conical and steamed corn-bread shape, which is produced from steamed high-quality sun-dried green teas. Its main origin is the Yunnan Province of China. Yunnan Bowl Tea is also called Xiaguan Bowl Tea since it was developed in Xiaguan, Yunnan. Similar to Pu’er Tea, Xiaguan Bowl Tea is a dark tea that can be divided into raw and ripe Bowl Teas. Raw Bowl Tea is compacted tea made directly from sun-dried green tea fermented by artificial heap fermentation [4]. Clinical studies have shown that Bowl Tea has a significant weight loss effect in people aged 40–50 years. More than 70% of the cases show that Bowl Tea significantly reduced the levels of triglyceride in the human body [5]. In some experiments, participants drank three cups of Bowl Tea per day for a month, with a decrease in the cholesterol levels in half of the subjects [6]. The lipid-lowering effect of Yunnan Bowl Tea is similar to that of the lipid-lowering drug, clofibrate, with a lipid-lowering rate of 64% [7]. However, there is still no definitive evidence to prove the specific mechanism of Bowl Tea on weight loss and lipid metabolism, and the specific efficacy components are unclear.

During the development of NAFLD, excessive free fatty acid deposition is the “first-hit”. Accumulated free fatty acid that injures the liver is the “second-hit” through reactive oxides, promoting the conversion of simple fatty liver disease to nonalcoholic steatohepatitis and cirrhosis [8]. Owing to the oxidation of a large number of free fatty acids and the activation of bypass pathways of fatty acid metabolism, excessive reactive oxygen species (ROS) are produced, resulting in hepatocyte injury and the activation of hepatic stellate cells (HSC). At the same time, the ability of steatotic hepatocytes to remove intestinal endotoxins is lowered, and fat tissue is stimulated by endotoxins to produce inflammatory factors that further enhance insulin resistance and HSC activation and promote the occurrence of hepatic fibrosis [9]. 

The intestinal tract plays an important role in the development of NAFLD. The absorption of long-chain fatty acids (LCFA) in the main components after fat digestion is closely related to the structural integrity and function of the small intestine. The weakening of the intestinal mucosal barrier function allows more bacterial endotoxins to diffuse into the blood circulation, causing liver cell damage through a series of inflammatory reactions [10]. The occurrence of NAFLD is also closely related to the changes in intestinal flora. Intestinal harmful bacteria, typically flagellated Gram-negative bacteria, increase in NAFLD patients, which will then be accompanied by the changes in the intestinal permeability and a systemic low-grade chronic inflammatory response [11]. In addition, studies have shown that the disorder of the intestinal flora will lead to increased absorption of free fatty acids and reduced heat production, which will eventually cause NAFLD by increasing the amount of stored fat [12]. Studies have shown that tea polyphenols have a good antioxidant effect and an excellent regulatory efficacy on the NAFLD-induced oxidative level in rats, thus protecting the liver tissue of rats [13]. Studies have shown that the polyphenol components contained in Bowl tea are different from those in green tea [14]. Bowl tea and green tea polyphenols may have different mechanisms on NAFLD, which is also an important topic in this study.

We observed the effect of Raw Bowl Tea polyphenols (RBTP) on NAFLD by inducing NAFLD in mice fed a high fat diet. The serum and liver tissues of NAFLD mice were analyzed, and the effects of RBTP on the markers of NAFLD were examined. At the same time, the effects of RBTP on the intestinal function and intestinal microecology of mice were further observed, and the role of RBTP in protecting the liver by regulating intestinal function and intestinal microecology was confirmed. In addition, the mechanism of RBTP was further elucidated by the composition analysis. This study comprehensively explained the mechanism of RBTP in preventing NAFLD by regulating intestinal function and microecology with its effective compounds. This study accumulates the theoretical basis for the further rational application of RBTP and lays the foundation for future clinical research.

## 2. Materials and Methods 

### 2.1. Extraction of RBTP

A 500 g raw Bowl tea (RBT) sample was crushed into powder, and 50 mL 45% (volume ratio) ethanol solution was added to the RBT powder and extracted at 90 °C for 30 min. After a repeated extraction, two extracts were combined, and the pH of the total extract was adjusted to 6.0. Then 800 mL mixed precipitant of AlCl_3_ (30 g, Tianjin Damao Chemical Reagent Factory, Tianjin, China) and ZnCl_2_ (60 g, Tianjin Damao Chemical Reagent Factory, Tianjin, China) was added to the extract for precipitation, and then the mixture was centrifuged at 3000× *g* for 10 min. 1000 mL hydrochloric acid (12%, volume ratio, Tianjin Damao Chemical Reagent Factory, Tianjin, China) was added to the collected precipitation for transsolution. The supernatant was separated, and 50 mL ethyl acetate (Tianjin Damao Chemical Reagent Factory, Tianjin, China) was added twice for extraction. Finally, the extract was subjected to rotary evaporation to obtain RBTP [15].

### 2.2. Determination of RBTP Composition

Two mL of chromatographic grade methanol were added separately to the following polyphenolic compounds: (-)-epicatechin gallate (ECG), gallic acid, (-)-epigallocatechin (EGC), caffeine, (-)-epigallocatechin gallate (EGCG), (-)-gallocatechin gallate (GCG), L-epicatechin (EC), and catechin standards. Each accurately weighed reference substance was fully dissolved by oscillation to obtain the standard solution. 10 mL of chromatographic grade methanol was added to accurately weighed 5 mg dried tea polyphenol extract, and was dissolved by oscillation. Samples were filtered with a microporous membrane (0.22 μm) to obtain the test solution. Component analysis was carried out under the following chromatographic conditions: Mobile phase A was methanol; mobile phase B was 0.1% formic acid; mobile phase C was acetonitrile; the flow rate was set at 0.6 mL/min; chromatographic column was Accucore PFP (2.6 μm, 50 × 2.1 mm); the column temperature was 30 °C; wavelength was 280 nm; injection volume was 10 μL. At the same time, the chromatographic peak area of each component was recorded to analyze the content of each component (Ultimate3000; Thermo Fisher Scientific, Inc., Waltham, MA, USA).

### 2.3. Culture and Induced Differentiation of 3T3-L1 Preadipocytes

3T3-L1 preadipocytes (American Type Culture Collection, Manassas, VA, USA) were cultured with DMEM (Thermo Fisher Scientific, Waltham, MA, USA) containing 10% calf serum at 37 °C and 5% CO_2_. When the cells were in good condition, they were inoculated on the culture plate and cultured for 48 h with DMEM containing 0.5 mmol/L isobutyl-3-methylxanthine, 0.25 μmol/L dexamethasone, 10 μg/mL insulin and 10% fetal bovine serum. Subsequently, DMEM medium containing 10% fetal bovine serum was used for further culture. The medium was changed every 2 days. After 8–12 days of differentiation, more than 85% of 3T3-L1 cells showed adipocyte phenotypes, which could be used in the experiment.

### 2.4. Effect of RBTP on the Proliferation of 3T3-L1 Preadipocytes Detected by XTT Assay

The 3T3-L1 preadipocytes were inoculated into 96-well plates at a cell concentration of 1.5 × 10^4^/mL, 100 μL medium was added to each well. After cell adherence, 200 μg/mL of RBTP, ECG, gallic acid, EGC, caffeine, EGCG, GCG, EC and catechin were added to intervention culture for 72 h. OD_570_ values of each group were detected by the XTT method, and the number of cells was calculated.

### 2.5. Establishment of NAFLD Mouse Model

Fifty 8-week-old SPF grade C57BL/6N mice (half male and half female, Chongqing Medical University, Chongqing, China) were fed for 1 w to adapt to the environment, and were divided into five groups on average: Normal group, model group, RBTP low-concentration gavage group (RBTP-L group), RBTP high-concentration gavage group (RBTP-H group), and bezafibrate gavage group (bezafibrate group), with 10 mice in each group. Mice in the normal group were fed normal maintenance foods and drinking water; mice in the other groups were fed a D12492 high-fat diet, while mice in the RBTP-L and RBTP-H groups were intragastrically administered with RBTP at the concentrations of 50 and 100 mg/kg once a day, respectively. Mice in the bezafibrate group were intragastrically administered with bezafibrate at the concentration of 100 mg/kg daily. After 12 w, all mice fasted for 24 h and then were sacrificed by cervical dislocation [16]. Blood was taken from the heart and liver; epididymal fat and small intestine tissues were collected for subsequent experiments. The liver tissue was weighed and a liver index was calculated by the formula: Liver weight (g)/mouse body weight (kg) × 100. This study was conducted in accordance with the Declaration of Helsinki, and the protocol was approved by the Ethics Committee of Chongqing Collaborative Innovation Center for Functional Food (201807004B), Chongqing, China.

### 2.6. Determination of Serum Biochemical Indicators of ALT, AST, AKP, TC, TG, LDL-C, D-LA, DAO, and LPS

The obtained mouse blood was centrifuged at 4000× *g* for 10 min, and the supernatant was collected. The serum levels of ALT, AST, AKP, TC, TG, HDL-C, LDL-C, D-LA, DAO, and LPS of the mice were determined by kit instructions (Nanjing Jiancheng Bioengineering Institute, Nanjing, Jiangsu, China).

### 2.7. Determination of Liver Tissue Biochemical Indicators of D-LA, DAO, and LPS

Nine mL of cold saline was added to 1 g of liver tissue, which was homogenized until no fibrous granules were found, and the supernatant was obtained after centrifugation (4000× *g*). The liver tissue levels of D-LA, DAO, and LPS of the mice were determined by kit instructions (Nanjing Jiancheng Bioengineering Institute, Nanjing, Jiangsu, China).

### 2.8. Determination of Liver Tissue of ROS

One g of liver tissue was suspended in buffer containing 20 mmol/L Tir-HCl (pH 7.4), 20 mmol/L NaH_2_PO_4_, 5 mmol/L MgCl_2_, 130 mmol/L KCl, and 30 mmol/L glucose, ground uniformly, centrifuged at 4 °C, 6000 rpm for 15 min, and the supernatant was removed. DCFH-DA (2’, 7’-dichlorodihydrofluorescein diacetate) was added to the supernatant and incubated at 37 °C for 15 min, then the reaction was terminated by adding 1 μmol/L of H_2_O_2_. The absorbance value was determined by fluorescence spectrophotometer (Evolution 220, Thermo Fisher Scientific, Waltham, MA, USA), and the relative level of ROS was expressed by fluorescence signal intensity.

### 2.9. Determination of Serum Cytokines IL-1β, IL-4, IL-6, IL-10, TNF-α, and INF-γ

The obtained mouse blood was centrifuged at 4000× *g* for 10 min, and the supernatant was collected. The serum levels of cytokines IL-6, IL-1β, TNF-α, INF-γ, IL-4, and IL-10 were determined by kit instructions (Abcam, Cambridge, MA, USA).

### 2.10. Pathological Observation of Liver, Epididymal Fat, and Small Intestine Tissues

Liver, epididymal fat, and the posterior part of the small intestine tissue at the size of 0.5 cm^2^ were collected and fixed in 10% formalin solution for 48 h. The liver and epididymal fat tissues were dehydrated, cleared, waxed, embedded, sectioned, and stained with H&E. The morphological changes were observed under optical microscope (BX43; Olympus, Tokyo, Japan).

### 2.11. Quantitative PCR (qPCR) Assay

The liver tissue, small intestine tissue, and feces of the mice were pulverized. RNAzol (Invitrogen, Carlsbad, CA, USA) was used to extract total RNA from the sample, and then the concentration of extracted total RNA was diluted to 1 μg/μL. 5 μL of the diluted total RNA solution was removed for reverse transcription, which was carried out according to the instruction of reverse transcription kit to obtain the cDNA template. 2 μL of cDNA template was mixed with 10 μL of SYBR Green PCR Master Mix (Thermo Fisher Scientific) and 1 μL upstream and downstream primers (Table 1). The system was reacted at 95 °C for 60 s; then at the conditions of 95 °C for 15 s, 55 °C for 30 s, 72 °C for 35 s, for 40 cycles. Finally, the DNA was detected at 95 °C for 30 s and 5 °C for 35 s; GAPDH was used as the internal reference (StepOnePlus Real-Time PCR System; Thermo Fisher Scientific). The 2^−ΔΔCt^ method was used to determine the level of relative gene expression [17].

### 2.12. Western Blot Analysis

100 mg of each liver and epididymal fat tissue samples were homogenized with 1 mL of RIPA and 10 μL of PMSF, then centrifuged at 12,000× *g* at 4 °C for 4 min. The intermediate protein layer solution was removed, and the BCA protein quantification kit was used for protein quantification. Samples of each group were diluted to 50 μg/mL, and the diluted protein was mixed with Sample Buffer at the ratio of 4:1 and heated at 100 °C for 5 min. Then Mixing Acrylamide, Resolving Buffer, Starcking Buffer, distilled water, 10% APS, and TEMED were mixed in proportion to make SDS-PAGE separation gel and stacking gel, and poured into the gel plate. The Prestained Protein Ladder and the sample were separately added into the sample wells of the gel plate, and the protein-loaded SDS-PAGE gel was subjected to vertical gel electrophoresis for 50 min. The polyvinylidene difluoride (PVDF) membrane was activated by methanol for 1 min and then transmembrane was performed. After that, the PVDF membrane was blocked by 5% fat-free milk containing TBST solution for 1 h. After blocking, the PVDF membrane was washed by TBST. The first antibody was incubated at 25 °C for 2 h, and the second antibody was incubated at 25 °C for 1 h. Finally, Supersignal West Pico PLUS was used to fill the PVDF membrane and was placed in the iBright FL1000 (Thermo Fisher Scientific) for observation [18].

### 2.13. Statistical Analysis

The serum and tissue assays of each mouse were performed in three parallel experiments and the average value was calculated. SAS9.1 statistical software (Version, Company, City, Country) was used for data analysis and one-way ANOVA method was used to analyze whether there were significant differences among groups of data at the level of *p* < 0.05 [18].

## 3. Results

### 3.1. Composition Analysis of RBTP

**Figure 1:**
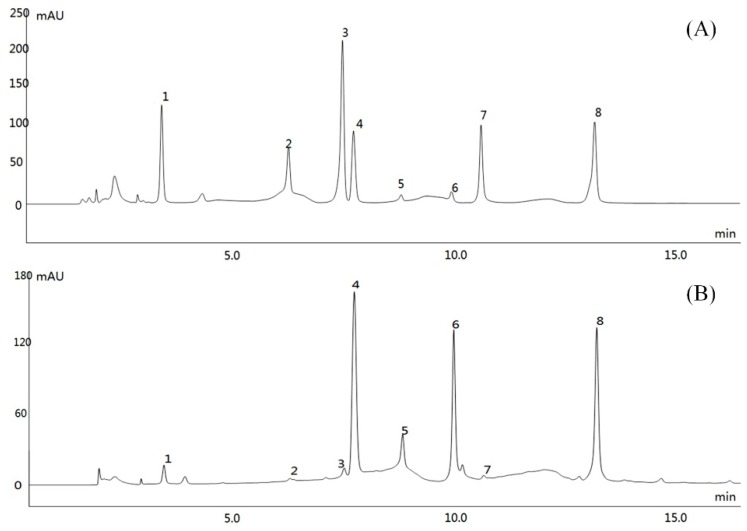
Polyphenol constituents of raw Bowl tea. (**A**) Standard chromatograms; (**B**) Polyphenols of raw Bowl tea chromatograms. 1: gallic acid, 2: (-)-epigallocatechin (EGC); 3: catechin; 4: caffeine; 5: L-epicatechin (EC); 6: (-)-epigallocatechin gallate (EGCG); 7: (-)-gallocatechin gallate (GCG); 8: (-)-epicatechin gallate (ECG).

HPLC analysis showed that RBTP contained seven polyphenolic compounds (Figure 1), which were gallic acid, (-)-epigallocatechin (EGC), catechin, L-epicatechin (EC), (-)-epigallocatechin gallate (EGCG), (-)-gallocatechin gallate (GCG), and (-)-epicatechin gallate (ECG). Because caffeine was not easily separated in the extraction of natural polyphenols, a small amount of caffeine remained in RBTP. Quantitative analysis showed that the sequence of RBTP polyphenol contents were as following: ECG > EGC > EGCG > catechin > gallic acid > EC = GCG > caffeine (Table 2).

### 3.2. Effect of Samples on Proliferation of 3T3-L1 Preadipocytes

The results showed that gallic acid, (-)-epigallocatechin, catechin, caffeine, L-epicatechin, (-)-epigallocatechin gallate, (-)-gallocatechin gallate, (-)-epicatechin gallate and RBTP could significantly (*p* < 0.05) inhibit the proliferation of 3T3-L1 preadipocytes (Table 3), and the effects of (-)-epigallocatechin and (-)-epigallocatechin gallate were better than those of RBTP, while the effects of other compounds were lower than that of RBTP.

### 3.3. Liver Index of Mice

Mice in the normal group had the lowest body weight, liver weight, and liver index, while those in the model group had the highest indices (Table 4). After the administration of RBTP and bezafibrate, the body weight, liver weight, and liver index of all NAFLD mice decreased. A high concentration of RBTP (RBTP-H) showed the most significant reducing effect on the body weight, liver weight, and liver index, making these indices close to those of the normal group.

### 3.4. Serum Levels of Biochemical Indicators of ALT, AST, AKP, TC, TG, HDL-C, and LDL-C in Mice

Table 5 indicates that the levels of ALT, AST, AKP, TC, TG, and LDL-C in the serum of healthy mice (normal group) were the lowest, while HDL-C levels were the highest. Mice with NAFLD (model group) showed a contrary trend. The serum levels of ALT, AST, AKP, TC, TG, and LDL-C were the highest and the HDL-C level was the lowest in the model group. After the administration of RBTP and bezafibrate, the levels of ALT, AST, AKP, TC, TG, and LDL-C in NAFLD mice decreased, while the level of HDL-C increased. The effect of RBTP-H was stronger than that of bezafibrate and RBTP-L.

### 3.5. Serum and Liver Tissue Levels of D-LA, DAO, and LPS in Mice

Table 6 and Table 7 show that the normal group mice had the lowest D-LA, DAO, and LPS serum levels, and the model group mice had the highest D-LA, DAO, and LPS levels. RBTP-H could reduce the D-LA, DAO, and LPS levels compared to the model group, and the mice in the RBTP-H group showed the lower D-LA, DAO, and LPS levels than mice in the bezafibrate and RBTP-L groups.

### 3.6. Serum Levels of Cytokines IL-1β, IL-4, IL-6, IL-10, TNF-α, and INF-γ in Mice

The serum cytokine detection assay showed that the serum levels of IL-1β, IL-4, IL-6, IL-10, TNF-α, and INF-γ in normal mice were the lowest (Table 8), while these indices in the model group were the highest. Both RBTP and bezafibrate could alleviate the effects of NAFLD on mice. The serum levels of cytokines IL-1β, IL-4, IL-6, IL-10, TNF-α, and INF-γin NAFLD mice (the model group) were significantly lower than those in mice of the model group without non-intervention (*p* < 0.05), and RBTP-H had the strongest action to decrease these cytokines, which was significantly stronger than the drug bezafibrate.

### 3.7. Liver Tissue Level ROS in Mice

**Figure 2:**
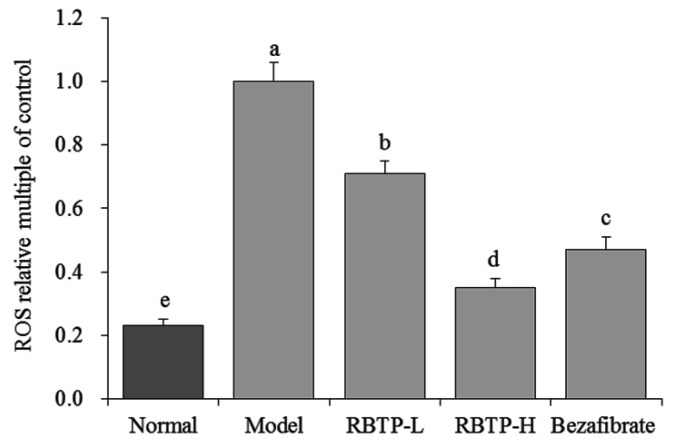
The ROS level of liver tissue in mice. ^a–e^ Mean values with different letters in the bar are significantly different (*p* < 0.05) according to Duncan’s multiple-range test. RBTP-L: mice treated with low concentrations of polyphenols of raw Bowl tea (50 mg/kg); RBTP-H: mice treated with high concentrations of polyphenols of raw Bowl tea (100 mg/kg); Bezafibrate: mice treated with bezafibrate (100 mg/kg).

The mice in model group showed the strongest ROS level and RBTP and bezafibrate could reduce the levels of ROS, with RBTP-H having better reducing effects than bezafibrate and RBTP-L (Figure 2).

### 3.8. Pathological Observation of Liver, Epididymis, and Small Intestine Tissue of Mice

**Figure 3:**
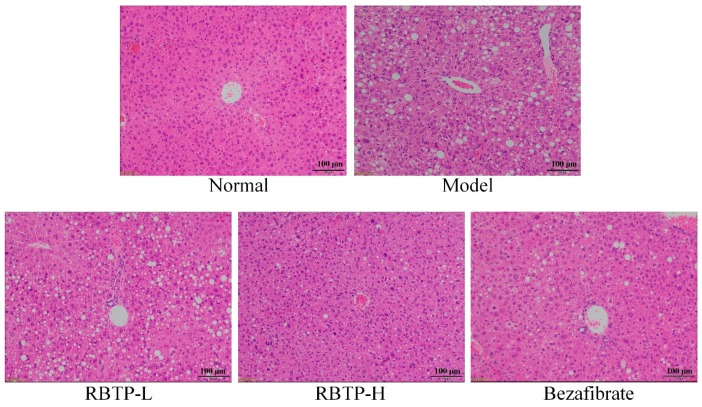
H&E pathological observation of hepatic tissue in mice. Magnification 100×. RBTP-L: mice treated with low concentrations of polyphenols of raw Bowl tea (50 mg/kg); RBTP-H: mice treated with high concentrations of polyphenols of raw Bowl tea (100 mg/kg); Bezafibrate: mice treated with bezafibrate (100 mg/kg).

The structure of hepatic lobules in normal mice was clear, the hepatocyte cord was arranged neatly, the cytoplasm of hepatocytes was observed to be fine granular, and there was no inflammatory cell infiltration in the portal canal area (Figure 3). The hepatocytes in mice of the model group had steatosis, and the lipid droplets in hepatocytes were diffuse. Granular accumulation could be seen, the lipid droplets increased obviously, and the inflammatory cells infiltrated significantly in interlobular and portal areas. In the RBTP-H, RBTP-L, and bezafibrate groups, the number of lipid droplets in the liver cells was significantly reduced, scattered, and sparse. The volume of lipid droplets was smaller, and the fat lesions were significantly alleviated. RBTP-H had stronger inhibitory effects on liver lesions than RBTP-L and bezafibrate did in NAFLD mice.

**Figure 4:**
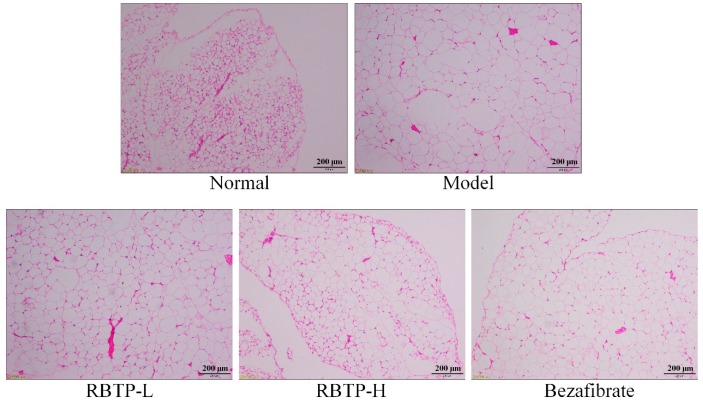
H&E pathological observation of epididymal tissue in mice. Magnification 100×. RBTP-L: mice treated with low concentrations of polyphenols of raw Bowl tea (50 mg/kg); RBTP-H: mice treated with high concentrations of polyphenols of raw Bowl tea (100 mg/kg); Bezafibrate: mice treated with bezafibrate (100 mg/kg).

Adipocytes in epididymal fat tissue of mice in the normal group were smaller and were arranged neatly (Figure 4). In the model group, adipocytes became larger and the cell membrane became thinner, with two adjacent cells merging into one cell. Adipose tissue of mice fed RBTP and bezafibrate was denser compared to the model group. After RBTP-H was administered to NAFLD mice, the size of epididymal fat cells was similar to that of the normal group, which could significantly reduce the fat cell hypertrophy caused by a high-fat diet. The effect was stronger than that of RBTP-L and bezafibrate.

**Figure 5:**
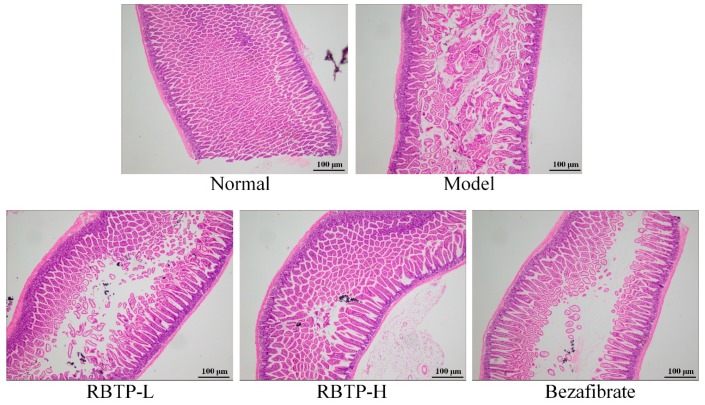
H&E pathological observation of small intestine tissue in mice. Magnification 100×. RBTP-L: mice treated with low concentrations of polyphenols of raw Bowl tea (50 mg/kg); RBTP-H: mice treated with high concentrations of polyphenols of raw Bowl tea (100 mg/kg); Bezafibrate: mice treated with bezafibrate (100 mg/kg).

Figure 5 shows no significant change in the morphology of intestinal mucosa in the normal group. The intestinal villi were arranged neatly, and there was no significant difference in mucosal epithelial thickness. In the model group, the height and width of villus decreased, and many villi were broken. Chronic inflammatory cell infiltration of monocytes and plasma cells appeared in the lamina propria of the intestinal mucosa of the model group. RBTP-H was able to minimize the damage of small intestine caused by NAFLD, and make the small intestine morphology closer to the normal group, which was better than the drug bezafibrate.

### 3.9. Expression of RNA and Protein in Mouse Liver Tissue

**Figure 6:**
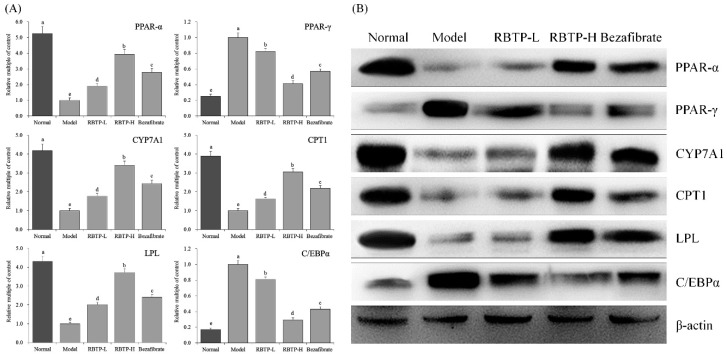
The PPAR-α, PPAR-γ, CYP7A1, CPT1, LPL, and C/EBPα mRNA (**A**) and protein (**B**) expression in hepatic tissue of mice. ^a–e^ Mean values with different letters in the bar are significantly different (*p* < 0.05) according to Duncan’s multiple-range test. RBTP-L: mice treated with low concentrations of polyphenols of raw Bowl tea (50 mg/kg); RBTP-H: mice treated with high concentrations of polyphenols of raw Bowl tea (100 mg/kg); Bezafibrate: mice treated with bezafibrate (100 mg/kg).

The mRNA and protein expressions of LPL, PPAR-α, CYP7A1, and CPT1 were strongest in the liver tissues of normal mice, while the expression intensity of PPAR-γ and C/EBP-α was weakest (Figure 6). The expression of LPL, PPAR-α, CYP7A1, and CPT1 in liver tissue was significantly decreased (*p* < 0.05), while the expression of PPAR-γ and C/EBP-α was significantly increased (*p* < 0.05) in mice induced by NAFLD. RBTP and bezafibrate were able to significantly inhibit the down-regulation of LPL, PPAR-α, CYP7A1, and CPT1 expression and the up-regulation of PPAR-γ and C/EBP-α in liver tissue of NAFLD mice (*p* < 0.05). The effect of RBTP-H was stronger and the expressions of PPAR-α, PPAR-γ, CYP7A1, CPT1, LPL, and C/EBPα in liver tissues of RBTP-H mice were close to those of normal mice.

### 3.10. Expression of RNA and Protein in Mice Small Intestine Tissue

**Figure 7:**
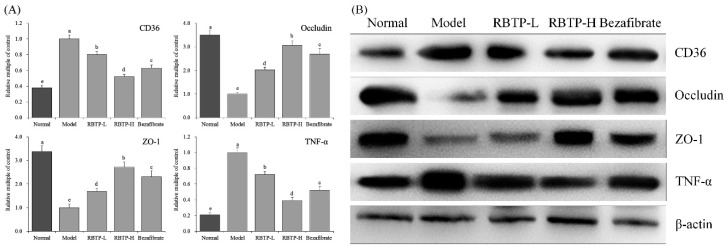
The CD36, occludin, ZO-1 and TNF-α mRNA (**A**) and protein (**B**) expression in small intestine tissue of mice. ^a–e^ Mean values with different letters in the bar are significantly different (*p* < 0.05) according to Duncan’s multiple-range test. RBTP-L: mice treated with low concentrations of polyphenols of raw Bowl tea (50 mg/kg); RBTP-H: mice treated with high concentrations of polyphenols of raw Bowl tea (100 mg/kg); Bezafibrate: mice treated with bezafibrate (100 mg/kg).

Figure 7 indicates that the mRNA and protein expressions of occludin and ZO-1 in the small intestines of the model group were significantly (*p* < 0.05) lower than those of other groups, while the expression of CD36 and TNF-α were significantly stronger. RBTP and bezafibrate could significantly up-regulate occludin and ZO-1 expression, and down-regulate CD36 and TNF-α expressions in the small intestines of mice in the model group. RBTP-H was more effective than RBTP-L and bezafibrate. The effect of RBTP-H could make the expression of CD36, occludin, ZO-1, and TNF-α in the small intestines of NAFLD mice close to those of normal mice.

### 3.11. Expression of Microbial RNA in Mice Feces

**Figure 8:**
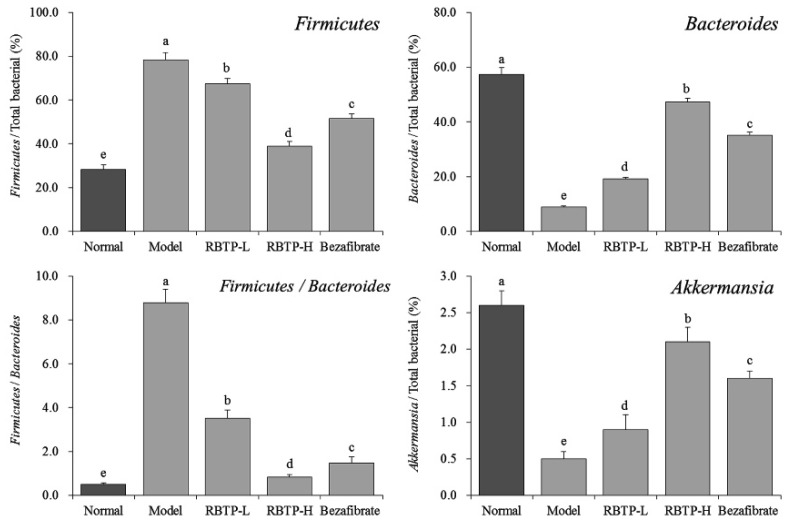
The mRNA expression in microorganisms in feces of mice. ^a–e^ Mean values with different letters in the bar are significantly different (*p* < 0.05) according to Duncan’s multiple-range test. RBTP-L: mice treated with low concentrations of polyphenols of raw Bowl tea (50 mg/kg); RBTP-H: mice treated with high concentrations of polyphenols of raw Bowl tea (100 mg/kg); Bezafibrate: mice treated with bezafibrate (100 mg/kg).

The Firmicutes level in the feces of the model group was the highest, while the Bacteroides and Akkermansia levels were the lowest (Figure 8), and the proportion of *Firmicutes*/*Bacteroides* was also significantly higher than that of the other groups (*p* < 0.05). The levels of *Firmicutes* were decreased in the feces of RBTP-H, RBTP-L and bezafibrate mice, the levels of *Bacteroides* and *Akkermansia* increased, and the proportion of *Firmicutes*/*Bacteroides* also decreased significantly (*p* < 0.05). At the same time, RBTP-H showed stronger decreasing effects on the level of *Firmicutes*, stronger increasing levels of *Bacteroides* and *Akkermansia*, and a more significant reducing effect on the ratio of *Firmicutes*/*Bacteroides* in the feces of NAFLD mice compared to RBTP-L and bezafibrate, which made the microbial status in the feces close to healthy mice (normal group).

## 4. Discussion

In recent years, the incidence of various metabolic syndromes have increased every year with the rapid increase of the obese population globally. The incidence of NAFLD is 6–45% in the general population, however, it can be as high as 90% in the severe obesity population [19]. NAFLD has become the most common cause of chronic liver disease in developed countries. Studies have shown that intestinal flora can cause intestinal dysfunction and abnormal lipid metabolism, which is closely related to the occurrence and development of NAFLD [20]. The intestinal flora of mice fed a high-sugar diet was also changed, showing a decrease in the number of Firmicutes and Bacteroides. High fructose intake could promote fat re-synthesis and inhibit fatty acid β oxidation, leading to hepatic steatosis and the initiation of inflammatory reaction and, ultimately, the development of NAFLD [21,22]. In this study, we found that, in contrast to polyphenols extracted from green tea, those extracted from Tuocha tea have a preventive effect against nonalcoholic fatty liver disease. 

Obesity is the main cause of nonalcoholic fatty liver disease. Thirty to fifty percent of obese people have fatty liver. Fatty liver is usually a reversible disease, and early prevention and diagnosis can normalize the liver [23]. Organ quality changes can directly reflect the body’s obesity. A long-term high-fat diet can lead to a stress reaction in the body, liver lipid accumulation, and other phenomena, leading to hepatomegaly and damaged liver function. ALT and AST are mainly distributed in hepatocytes. When hepatocyte necrosis occurs, ALT and AST are released into the blood circulation, causing serum enzymes to rise; the level is positively correlated with the degree of abnormal liver tissue [24,25]. Blood lipid levels can reflect the whole body’s lipid metabolism, and TG, TC, HDL-c, and LDL-c are hallmarks of blood lipids [25]. By exploring the regulatory effect of RBTP on NAFLD and determining the degree of liver enlargement in mice by the liver organ index, it is also possible to determine hepatomegaly, liver function, and abnormal blood lipids caused by NAFLD by measuring the markers in the serum. In this study, RBTP effectively lowered abnormal liver enlargement and abnormal serum index, with the alleviation of NAFLD.

Fat accumulation caused by abnormal lipid metabolism induced by NAFLD can also cause chronic tissue inflammation, speeding up the process of fat accumulation and insulin resistance, thus reflecting the abnormal immune system in the body. The accumulation of lipids in the cytoplasm of liver cells (the first hit) triggers a series of cytotoxic events (the second hit), leading to an inflammatory response in the liver. The occurrence and progress of NAFLD are mainly related to insulin and leptin resistance, the production of free radicals, excessive accumulation of visceral fat, and inflammation of adipose tissue and liver tissue [26]. NAFLD causes abnormalities in IL-1beta, IL-4, IL-6, IL-10, TNF-alpha and INF-gamma cytokines. In this study, RBTP also inhibited cytokine abnormalities caused by NAFLD [27,28,29].

Studies have shown that NAFLD increases the risk of intestinal inflammation, leading to a significant increase in intestinal permeability during intestinal inflammation [30,31]. D-lactic acid is the product of inherent bacteria in gastrointestinal tract. Measuring the level of D-lactic acid in blood can reflect the integrity of intestinal mucosa and the change of intestinal mucosal permeability [32]. LPS may be involved in the inflammatory response of the liver and the accumulation of fat in the liver, which is closely related to the formation of NAFLD [33]. This study also showed that RBTP could reduce the increase of intestinal permeability caused by NAFLD, alleviate intestinal injury, and avoid intestinal dysfunction caused by NAFLD. Additionally, RBTP also could reduce fat accumulation through reducing LPS.

The production of reactive oxygen species (ROS) in the liver exceeds the scavenging capacity of the antioxidant system, resulting in oxygen stress. When oxygen stress increases, ROS reacts with unsaturated fatty acids of membrane phospholipids to form lipid peroxide (LPO), which leads to inflammation, necrosis, and fibrosis [34]. In this study, RBTP has also been demonstrated to alleviate the effects of oxidative stress, protecting the liver and reducing NAFLD by lowering ROS levels in liver tissue.

Peroxisome proliferator-activated receptor (PPAR) is a member of the nuclear receptor transcription factor superfamily that regulates the expression of target genes [35]. PPAR-γ is mainly expressed in adipose tissue, which is related to the molecular mechanism of adipocyte overdifferentiation and adipocyte formation. PPAR-γ not only regulates the expression of genes related to lipid metabolism, but also is the main regulator of gene expression in adipocytes and signal transduction in insulin cells. C/EBP-α is a transcription factor that plays an important role in adipocyte differentiation and directly regulates adipocyte differentiation. In addition, there is a synergistic effect between C/EBP-α and PPAR-γ. The activation of PPAR-γ can induce the expression of C/EBP-α gene, and C/EBP-α has a positive feedback effect on PPAR-γ [36,37]. 

Carnitine palmitoyl transferase (CPT1) is an important rate-limiting enzyme in the process of fatty acid oxidation (FAO). The level of CPT1 is closely related to the occurrence of hyperlipidemia [38,39]. PPAR-α is an upstream transcription factor in fatty acid oxidation. CPT-1 is a key downstream target gene. The level of CPT-1 expression in the liver is regulated by its upstream factor PPAR-α, and the PPAR-α/CPT-1 combination is an important pathway in liver lipid metabolism. As a receptor for free fatty acids, PPAR regulates the body’s lipid metabolism. PPAR-α accelerates the transport of fatty acids to mitochondria by inducing the expression of specific CPT1 in muscle and liver, and finally promotes the β oxidation of fatty acids [40,41]. Lipoprotein lipase (LPL) is a proteolytic enzyme and a key enzyme in lipid metabolism pathway. Its main function is to catalyze the decomposition of triglyceride (TG) in chyle particles (CM) and very low density lipoprotein (VLDL) in plasma into free fatty acids and to promote the transport of proteins, phospholipids, and apolipoproteins, thus promoting the level of high density lipoproteins (HDL) [42,43]. Nearly 50% of cholesterol in human body is converted into bile acid by the catalysis of CYP7A1. Therefore, the CYP7A1 gene, as the most important regulatory gene in cholesterol synthesis, plays an important role in maintaining cholesterol homeostasis and bile acid synthesis [44,45]. In this study, fat accumulation occurred in the liver of mice in the NAFLD model group. RBTP up-regulated the mRNA and protein expression of LPL, PPAR-α, CYP7A1, and CPT1 in the liver of NAFLD mice and down-regulated the expression of PPAR-γ and C/EBP-α, thus reducing lipid accumulation in mice induced by high-fat diet and preventing NAFLD.

Intestinal mucosal barrier dysfunction is common in liver diseases. Studies have shown that when NAFLD occurs, intestinal flora alters, intestinal mucosal permeability increases, and the incidence of intestinal endotoxemia becomes high [46]. The anatomical basis of intestinal mucosal epithelial barrier function originates from the tight junctions (TJs) between intestinal epithelial cells. TJs are composed of a variety of tight junction proteins such as ZO-1 and occludin, which play important roles in maintaining epithelial cell polarity and regulating intestinal permeability. Abnormal expression of these tight junction proteins or abnormal distribution of tight junction proteins due to changes in the cytoskeletal structure can lead to destruction of TJs [47]. Clinical studies have shown that the expression of ZO-1 protein in duodenal crypts and intestinal villi in patients with NAFLD is significantly lower than that in normal individuals, and is closely related to increased intestinal permeability and intestinal bacterial overgrowth [41]. Animal experiments have also shown that the expression of ZO-1 and occludin in the small intestine of NAFLD rats is also significantly reduced [48]. These results suggest that the impaired intestinal barrier function in NAFLD may be related to the decreased expression of tight junction proteins. 

Lipid deposition in the liver plays a crucial role in the development of NAFLD. Increased intake of dietary fats, especially LCFA, is an important part of free fatty acid intake of human body. Fatty acid transposase CD36, the membrane protein of small intestinal absorptive cells, is located in the brush border membrane of small intestine villus cells and is closely related to LCFA absorption [49]. The expression of TNF-α in small intestinal cells of the NAFLD model is elevated, which promotes the expression of iNOS and increases the synthesis of nitric oxide in small intestinal cells [50]. Increased TNF-α and NO can down-regulate the expression of tight junction proteins in the small intestinal mucosa, resulting in the destruction of tight junction structures, increased the permeability of the small intestinal mucosa, as well as promoting diffusion of the endotoxins produced by the intestinal Gram-negative bacteria [51]. RBTP prevented abnormal protein expressions induced by NAFLD in the small intestine of mice. The effect of RBTP at high concentration was significant and superior to the clinical drug bezafibrate.

Present in-depth research has revealed that intestinal flora disorders play a significant role in the occurrence and development of the nutritional metabolic disease NAFLD [52]. Long-term high-fat and high-sugar diets lead to intestinal flora disorder, which induces the production of harmful bacteria, especially flagellated Gram-negative bacteria, leading to excessive LPS production. LPS enters the blood circulation through damaged intestinal epithelial cells, causing systemic low-grade chronic inflammation in various tissues and organs of the body. When liver dysfunction occurs, the disorder of lipid metabolism leads to fat accumulation, significant fat infiltration in the liver, increased body mass, and impairment of glucose and lipid metabolism [53]. Studies have shown that the abundance of harmful bacteria represented by Firmicutes is reduced and the abundance of beneficial bacteria represented by Bacteroides is increased by improving the disorder of intestinal flora in the development of NAFLD. At the same time, the abundance of bacterial Akkermansia is increased, which significantly inhibits the occurrence and development of NAFLD. Fat accumulation in the liver can be significantly reduced by regulating the proportion of the intestinal microorganisms mentioned above, thereby generally alleviating the pathological symptoms of NAFLD [54]. In this study, we showed that the RBTP gavage could also improve the status of intestinal microorganisms in NAFLD mice and play a role in preventing NAFLD.

Catechin regulates the energy balance of the body, reduces oxidative stress and inflammation, and plays an important role in protecting body tissues in the progression of NAFLD. Catechin can alleviate the degree of hepatic steatosis by reducing adipogenesis and enhancing the antioxidant defense ability of liver in obese rats [55]. Clinical studies have shown that EGCG can reduce ALT, TG, and atherosclerotic lipoprotein levels, which lowers the incidence of cardiovascular disease associated with NAFLD [56]. Animal experiments show that gallic acid can reduce fat in obese mice; therefore, it is also reasonable to prevent NAFLD through its lipid-lowering effect [57]. EC, EGC, GCG, and ECG are all active substances with reduced fat-lowering effects, which can prevent cardiovascular diseases and other diseases caused by abnormal lipid metabolism. These active substances can also prevent NAFLD by regulating lipid metabolism [58,59]. Caffeine is not a polyphenolic compound, however, and is difficult to separate from tea polyphenols. When extracting polyphenols from tea leaves, caffeine often coexists with tea polyphenols. Caffeine has the efficacy of increasing the concentration of fatty acids in the blood. Once the concentration of fatty acids increases, the fatty acids are absorbed by the muscles and generate energy, thereby promoting the decomposition of accumulated fat in the body. Therefore, caffeine can also prevent NAFLD by reducing fat [60]. Tea polyphenols are converted into small molecular phenolic acids by the action of intestinal microbes and then are methylated, glucuronated, sulfated, or nucleated into the blood. The role of RBLP may be a combination of many substances. In this study, cell experiments showed that the above compounds monomers could significantly reduce the proliferation of 3T3-L1 preadipocytes. However, at the same concentration, (-)-epicatechin gallate, as the component with the highest RBTP content, was not as effective as mixture RBTP. Among the components contained in RBTP, at the same concentration, only two compounds ((-)-epigallocatechin and (-)-epigallocatechin gallate) were more effective than RBTP. These two components account for only 15.5% of RBTP content, but they played the most important role in the inhibition of RBTP on adipocytes. Therefore, in further animal experiments, the two components of RBTP should play a central role in NAFLD prevention. Of course, RBLP as a natural substance, in addition to the individual role of compounds, contains a variety of compounds may also produce a certain synergistic effect, enhancing the effect.

Valerolactone is converted into valeric acid by isomerization, and is oxidized or glycinated into phenylacetic, benzoic, hydroxypropionic, and hippuric acids in the liver [61]. The bioavailability of tea polyphenols is improved after they are transformed into small molecular substances by intestinal microorganisms. Under the action of microbes, polyphenols are metabolized into nutrients that can be directly absorbed and affect the lipid metabolism in the intestinal tract of animals [62]. On the other hand, polyphenols can regulate the composition of intestinal microorganisms, especially promote the growth of *Bifidobacterium* and reduce the proportion of *Firmicutes*/*Bacteroidetes*. Drinking tea can increase the proportion of *Bifidobacterium* in the intestinal tract, promote the development of beneficial intestinal microorganisms, and improving health. Polyphenols regulate the proportion of intestinal microorganisms and the absorption of fatty acids, control lipid metabolism, and play a role in preventing NAFLD [10,62]. Nuclear receptor (NRS), which is a transcription factor activated by ligands, is the main regulator of the metabolism of nutrients in the body. NRS can regulate many transcription factors including PPARs, thus regulating NAFLD [63]. It plays an important role in the prevention of NAFLD. In further research, we will also study the mechanism of RBTP in preventing NAFLD by regulating NRS.

## 5. Conclusions

This study investigated the preventive effect of RBTP on NAFLD in mice. RBTP effectively improved the imbalanced lipid metabolism in the serum and liver tissue, and also reduced the hepatic inflammatory response in NAFLD. RBTP also inhibited the injury of the small intestine, improved the intestinal environment, inhibited harmful bacteria and increased the number of beneficial bacteria, and prevented NAFLD by regulating intestinal function. This study has built the foundation for further research on RBTP. Since only animal studies were carried out in this study, future human clinical trials will be needed to confirm the preventive effect of RBTP on NAFLD.

## Figures and Tables

**Table 1:** Sequences of primers used in this study.

Gene Name	Sequence
PPAR-α	Forward: 5’-CCTCAGGGTACCACTACGGAGT-3’
	Reverse: 5’-GCCGAATAGTTCGCCGAA-3’
PPAR-γ	Forward: 5’-AGGCCGAGAAGGAGAAGCTGTTG-3’
	Reverse: 5’-TGGCCACCTCTTTGCTGTGCTC-3’
CYP7A1	Forward: 5’-AGCAACTAAACAACCTGCCAGTACTA-3’
	Reverse: 5’-GTCCGGATATTCAAGGATGCA-3’
CPT1	Forward: 5’-AAAGATCAATCGGACCCTAGACA-3’
	Reverse: 5’-CAGCGAGTAGCGCATAGTCA-3’
LPL	Forward: 5’-AGGGCTCTGCCTGAGTTGTA-3’
	Reverse: 5’-AGAAATCTCGAAGGCCTGGT-3’
C/EBPα	Forward: 5’-TGGACAAGAACAGCAACGAGTAC-3’
	Reverse: 5’-GCAGTTGCCCATGGCCTTGAC-3’
CD36	Forward: 5’-GATGACGTGGCAAAGAACAG-3’
	Reverse: 5’-TCCTCGGGGTCCTGAGTTAT-3’
Occludin	Forward: 5’-TGTGGATAAGGAACACATTTATGA-3’
	Reverse: 5’-CAGACACATTTTTAACCCACTCTTCA-3’
ZO-1	Forward: 5’-TGAACGCTCTCATAAGCTTCGTAA-3’
	Reverse: 5’-ACCGTACCAACCATCATTCATTG-3’
TNF-α	Forward: 5’-CAGGCGGTGCCTATGTCTC-3’
	Reverse: 5’-CGATCACCCCGAAGTTCAGTAG-3’
*Firmicutes*	Forward: 5’-TGAAACTYAAAGGAATTGACG-3’
	Reverse: 5’-ACCATGCACCACCTGTC-3’
*Bacteroides*	Forward: 5’-CRAACAGAATTAGATACCCT-3’
	Reverse: 5’-GGTAAGGTTCCTCGCGTAT-3’
*Akkermansia*	Forward: 5’-GGAGATTACTGCCCTGGCTCCTA-3’
	Reverse: 5’-CACTCATCGTACTCCTGCTTGTTGCTG-3’
GAPDH	Forward: 5’-ACCCAGAAGACTGTGGATGG-3’
	Reverse: 5’-ACACATTGGGGGTAGGAACA-3’

PPAR-α: peroxisome proliferator-activated receptor-alpha; PPAR-γ: peroxisome proliferator-activated receptor-gamma; CYP7A1: cholesterol 7 alpha-hydroxylase; CPT1: carnitine palmitoyltransferase 1; C/EBPα: CCAAT enhancer-binding protein alpha; CD36: cluster of differentiation 36; ZO-1: zonula occludens-1; TNF-α: tumor necrosis factor alpha; GAPDH: glyceraldehyde-3-phosphate dehydrogenase.

**Table 2:** Contents of polyphenols of small-leaved Kuding tea.

Polyphenol	Content (mg/g)
Gallic acid	15.0
(-)-epigallocatechin	107.5
Catechin	22.5
Caffeine	2.5
L-Epicatechin	7.5
(-)-epigallocatechin gallate	47.5
(-)-gallocatechin gallate	7.5
(-)-epicatechin gallate	230.0

**Table 3:** Effect of samples on proliferation of 3T3-L1 preadipocytes.

Sample (200 μg/mL)	Cell Counts (10^4^/mL)
Untreatment	14.63 ± 0.42 ^a^
RBTP	8.64 ± 0.53 ^d^
Gallic acid	12.71 ± 0.46 ^b^
(-)-epigallocatechin	7.44 ± 0.52 ^e^
Catechin	8.61 ± 0.36 ^d^
Caffeine	9.66 ± 0.50 ^c^
L-Epicatechin	9.29 ± 0.44 ^c^
(-)-epigallocatechin gallate	6.82 ± 0.45 ^f^
(-)-gallocatechin gallate	12.51 ± 0.58 ^b^
(-)-epicatechin gallate	9.62 ± 0.49 ^c^

Values presented are the mean ± standard deviation (*N* = 6/group). ^a–f^ Mean values with different letters over the same row are significantly different (*p* < 0.05) according to Duncan’s multiple range test. RBTP: polyphenol of raw Bowl tea.

**Table 4:** The liver index of mice with nonalcoholic fatty liver disease.

Group	Body Weight	Live Weight	Liver Index
Normal	31.15 ± 1.63 ^b^	1.37 ± 0.14 ^b^	4.40 ± 0.15 ^c^
Model	42.32 ± 2.79 ^a^	2.45 ± 0.52 ^a^	5.79 ± 0.35 ^a^
RBTP-L	40.85 ± 2.81 ^a^	2.12 ± 0.33 ^a^	5.19 ± 0.23 ^ab^
RBTP-H	34.60 ± 2.05 ^b^	1.67 ± 0.23 ^ab^	4.83 ± 0.26 ^b^
Bezafibrate	37.39 ± 2.14 ^ab^	1.86 ± 0.18 ^ab^	4.97 ± 0.18 ^ab^

Values presented are the mean ± standard deviation (*N* = 10/group). ^a–c^ Mean values with different letters over the same column are significantly different (*p* < 0.05) according to Duncan’s multiple range test. RBTP-L: mice treated with low concentrations of polyphenols of raw Bowl tea (50 mg/kg); RBTP-H: mice treated with high concentrations of polyphenols of raw Bowl tea (100 mg/kg); Bezafibrate: mice treated with bezafibrate (100 mg/kg).

**Table 5:** The levels of ALT, AST, AKP, TC, TG, HDL-C, and LDL-C in serum of mice.

Group	Normal	Model	RBTP-L	RBTP-H	Bezafibrate
ALT (U/L)	16.02 ± 1.14 ^e^	51.62 ± 2.45 ^a^	35.70 ± 1.73 ^b^	25.84 ± 1.66 ^d^	31.12 ± 1.52 ^c^
AST (U/L)	10.26 ± 2.31 ^e^	53.35 ± 3.15 ^a^	40.82 ± 2.74 ^b^	17.36 ± 1.89 ^d^	24.31 ± 2.30 ^c^
AKP (U/L)	33.52 ± 6.71 ^e^	87.36 ± 7.03 ^a^	63.05 ± 4.18 ^b^	45.96 ± 3.24 ^d^	56.37 ± 4.02 ^c^
TC (mmol/L)	1.87 ± 0.21 ^e^	5.32 ± 0.36 ^a^	4.01 ± 0.23 ^b^	2.57 ± 0.31 ^d^	3.11 ± 0.27 ^c^
TG (mmol/L)	0.55 ± 0.04 ^e^	1.62 ± 0.17 ^a^	1.23 ± 0.13 ^b^	0.71 ± 0.05 ^d^	0.92 ± 0.08 ^c^
HDL-C (mmol/L)	1.11 ± 0.15 ^a^	0.29 ± 0.05 ^e^	0.53 ± 0.06 ^d^	0.87 ± 0.05 ^b^	0.70 ± 0.04 ^c^
LDL-C (mmol/L)	0.47 ± 0.03 ^e^	1.33 ± 0.12 ^a^	1.01 ± 0.07 ^b^	0.63 ± 0.04 ^d^	0.88 ± 0.07 ^c^

Values presented are the mean ± standard deviation (*N* = 10/group). ^a–e^ Mean values with different letters over the same row are significantly different (*p* < 0.05) according to Duncan’s multiple range test. RBTP-L: mice treated with low concentrations of polyphenols of raw Bowl tea (50 mg/kg); RBTP-H: mice treated with high concentrations of polyphenols of raw Bowl tea (100 mg/kg); Bezafibrate: mice treated with bezafibrate (100 mg/kg). ALT: alanine aminotransferase; AST: aspartate aminotransferase; AKP: alkaline phosphatase; TC: total cholesterol; TG: triglyceride; HDL-C: high density lipoprotein cholesterol; LDL-C: low density lipoprotein cholesterol.

**Table 6:** The levels of D-LA, DAO, and LPS in serum of mice.

Group	Normal	Model	RBTP-L	RBTP-H	Bezafibrate
D-LA (mg/L)	0.41 ± 0.06 ^e^	1.48 ± 0.11 ^a^	1.08 ± 0.16 ^b^	0.62 ± 0.08 ^d^	0.81 ± 0.10 ^c^
DAO (pg/mL)	81.03 ± 6.32 ^e^	225.61 ± 11.03 ^a^	145.63 ± 8.97 ^b^	110.39 ± 5.44 ^d^	189.27 ± 12.52 ^c^
LPS (U/L)	8.63 ± 0.42 ^e^	17.11 ± 1.05 ^a^	15.36 ± 0.74 ^d^	10.29 ± 0.41 ^b^	12.05 ± 0.51 ^c^

Values presented are the mean ± standard deviation (*N* = 10/group). ^a–e^ Mean values with different letters over the same row are significantly different (*p* < 0.05) according to Duncan’s multiple range test. RBTP-L: mice treated with low concentrations of polyphenols of raw Bowl tea (50 mg/kg); RBTP-H: mice treated with high concentrations of polyphenols of raw Bowl tea (100 mg/kg); Bezafibrate: mice treated with bezafibrate (100 mg/kg). D-LA: D-Lactate; DAO: diamine oxidase; LPS: lipopolysaccharide.

**Table 7:** The levels of D-LA, DAO, and LPS in liver tissue of mice.

Group	Normal	Model	RBTP-L	RBTP-H	Bezafibrate
D-LA (mg/gprot)	0.26 ± 0.03 ^e^	0.63 ± 0.04 ^a^	0.53 ± 0.03 ^b^	0.37 ± 0.02 ^d^	0.44 ± 0.03 ^c^
DAO (pg/gprot)	12.51 ± 3.86 ^e^	59.71 ± 4.32 ^a^	48.12 ± 2.33 ^b^	20.36 ± 3.02 ^d^	31.08 ± 3.67 ^c^
LPS (U/gprot)	0.48 ± 0.04 ^e^	0.97 ± 0.05 ^a^	0.78 ± 0.05 ^d^	0.60 ± 0.04 ^b^	0.69 ± 0.03 ^c^

Values presented are the mean ± standard deviation (*N* = 10/group). ^a–e^ Mean values with different letters over the same row are significantly different (*p* < 0.05) according to Duncan’s multiple range test. RBTP-L: mice treated with low concentrations of polyphenol of raw Bowl tea (50 mg/kg); RBTP-H: mice treated with high concentrations of polyphenol of raw Bowl tea (100 mg/kg); Bezafibrate: mice treated with bezafibrate (100 mg/kg). D-LA: D-Lactate; DAO: diamine oxidase; LPS: lipopolysaccharide.

**Table 8:** The levels of IL-1β, IL-4, IL-6, IL-10, TNF-α and INF-γ in serum of mice.

Group	Normal	Model	RBTP-L	RBTP-H	Bezafibrate
IL-1β	202.51 ± 10.38 ^e^	544.39 ± 21.05 ^a^	410.81 ± 26.33 ^b^	272.82 ± 20.83 ^d^	342.08 ± 22.57 ^c^
IL-4	6.85 ± 0.31 ^e^	63.32 ± 2.34 ^a^	47.02 ± 3.01 ^b^	14.83 ± 1.01 ^d^	27.32 ± 1.24 ^c^
IL-6 (pg/mL)	43.10 ± 3.65 ^e^	145.98 ± 7.82 ^a^	114.29 ± 4.38 ^b^	67.08 ± 2.17 ^d^	85.33 ± 5.12 ^c^
IL-10 (pg/mL)	125.17 ± 8.32 ^e^	642.75 ± 23.85 ^a^	431.57 ± 16.35 ^b^	194.32 ± 9.33 ^d^	265.48 ± 13.37 ^c^
TNF-α (pg/mL)	20.85 ± 2.18 ^e^	117.63 ± 7.86 ^a^	83.19 ± 5.39 ^b^	34.57 ± 4.21 ^d^	50.28 ± 4.52 ^c^
INF-γ (pg/mL)	14.38 ± 1.71 ^e^	104.58 ± 6.63 ^a^	70.25 ± 3.88 ^b^	23.47 ± 2.20 ^d^	37.82 ± 3.17 ^c^

Values presented are the mean ± standard deviation (*N* = 10/group). ^a–e^ Mean values with different letters over the same row are significantly different (*p* < 0.05) according to Duncan’s multiple range test. RBTP-L: mice treated with low concentrations of polyphenols of raw Bowl tea (50 mg/kg); RBTP-H: mice treated with high concentrations of polyphenols of raw Bowl tea (100 mg/kg); Bezafibrate: mice treated with bezafibrate (100 mg/kg). IL-1β: interleukin 1 beta; IL-4: interleukin 4; IL-6: interleukin 6; IL-10: interleukin 10; TNF-α: tumor necrosis factor alpha; INF-γ: interferon gamma.
